# SbCl_3_-catalyzed one-pot synthesis of 4,4′-diaminotriarylmethanes under solvent-free conditions: Synthesis, characterization, and DFT studies

**DOI:** 10.3762/bjoc.7.19

**Published:** 2011-01-31

**Authors:** Ghasem Rezanejade Bardajee

**Affiliations:** 1 Department of Chemistry, Payame Noor University, Qazvin Branch, P.O. Box 878, Qazvin, Iran, Tel.: 982813336366, Fax: 982813344081

**Keywords:** diaminotriarylmethane, SbCl_3_, solvent-free reactions, synthesis, vibrational analysis

## Abstract

A simple, efficient, and mild procedure for a solvent-free one-step synthesis of various 4,4′-diaminotriarylmethane derivatives in the presence of antimony trichloride as catalyst is described. Triarylmethane derivatives were prepared in good to excellent yields and characterized by elemental analysis, FTIR, ^1^H and ^13^C NMR spectroscopic techniques. The structural and vibrational analysis were investigated by performing theoretical calculations at the HF and DFT levels of theory by standard 6-31G*, 6-31G*/B3LYP, and B3LYP/cc-pVDZ methods and good agreement was obtained between experimental and theoretical results.

## Introduction

The leuco forms of triarylmethine dyes are compounds with numerous diverse industrial, biological, and analytical applications. They have a broad spectrum of technological applications. For instance, they have been used not only in textile industry to dye wool, nylon, silk, leather, cotton, and polyacrylonitrile fibers, but they also have applications for coloring of plastics, varnishes, waxes, and oils. They have been used in novel types of colorless copying papers, pressure and heat-sensitive materials, light-sensitive papers, ultrasonic recording papers, electrothermic heat-sensitive recording papers, inks, crayons, typewritten ribbons, and photoimaging systems [1–3]. These compounds have been employed as dye precursors in nanocomposite preparations [4], in photoresponsive polymers [5], and as the thermal iniferter (initiator–transfer–terminator agent) in pseudo-living radical polymerizations [6]. They are also used in low-dose dosimeters [7], in chemical radiochromic dosimeters [8] and as sensitizers for photoconductivity [9].

The leuco forms of triarylmethine dyes are extensively used in biological applications. They show phototoxicity toward tumor cells [10] and also demonstrate antifungal [11–13], antitubercular [14], anti-infective, and antimicrobial activity [15]. Additionally, they have been used for sterilization of trypanosome cruizi-infected blood [16], in biotechnology process control [17–18], in dye-assisted laser inactivation of enzymes [19], in wastewater treatment plants [20], and in the photochemotherapy of neoplastic diseases [21–23]. In analytical chemistry, they are used as indicators in calorimetric and titrimetric determinations [1], in detection of various heavy metals [24], and for the detection of iodide [25] and carboxylic acids [26].

Diaminotriphenylmethine (DTM) dyes are the most important group of triarylmethine dyes and were selected for the present study due to their brilliance, high pictorial strength, low cost, and wide variety of applications. This group includes a broad range of dyes such as Cresol Red, Bromocresol Green, Light Green SF Yellowish, Victoria Blue BO, Ethyl Green, Brilliant Green, Diaminotriphenylmethane, Fast Green FCF, Green S, Fuchsine Acid, Chlorophenol Red, Crystal Violet Lactone, Fuchsine, Pararosaniline, Water Blue, Thymolphthalein, Bromocresol Purple, and Aurin. These compounds are usually soluble in non-polar organic solvents and are insoluble in water. Because of the wide range of applications of DTMs, the development of new and more efficient synthetic methods for their preparation is of importance.

Various procedures for the preparation of triarylmethane compounds can be found in the literature. For instance, they can be prepared by the palladium-catalyzed arylation of aryl(azaaryl)methanes with aryl halides [27], cationic Pd(II)/bipyridine-catalyzed addition of arylboronic acids to arylaldehydes [28], and by Friedel–Crafts type catalytic alkylation of aromatic rings with aromatic aldehydes and their imines [29–34].

The reaction of arylaldehydes with *N,N*-dimethylaniline is one of the most efficient methods for the synthesis of DTMs. This reaction is usually carried out in the presence of Brønsted acids such as sulfuric acid, hydrochloric acid, methanesulfonic acid, or *p*-TSA, as well as Lewis acids such as zinc chloride, zeolites, montmorillonite K-10, and polymer-supported sulfonic acid (NKC-9) [35–40]. Microwave-assisted synthesis of DTMs in the presence of aniline hydrochloride has also been described [41]. Recently, bismuth(III) nitrate and zirconium(IV) dichloride oxide octahydrate have been successfully used for the preparation of DTMs in our group [42–43].

The reported procedures are associated with certain limitations, such as low yields, the use of corrosive acids and excess solvent, severe reaction conditions, long reaction times, costly reagents, as well as the inconvenience in handling the reagents. Considering these restrictions, the development of new and simple synthetic methods for the efficient preparation of DTMs is therefore an interesting challenge. In this contribution, we describe a new route for the preparation of DTM derivatives under solvent-free conditions by the use of SbCl_3_ as a versatile catalyst. In addition, structural and vibrational studies of leuco compounds by DFT methods were investigated for the first time.

## Results and Discussion

### Synthesis of DTM

**Scheme 1:**
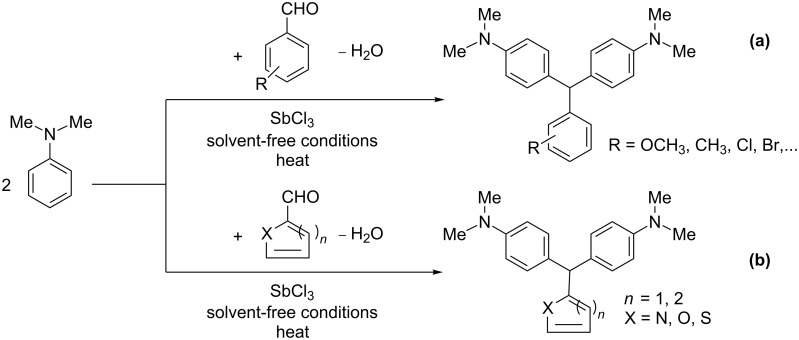
General route for the synthesis of 4,4’-diaminotriarylmethane derivatives in the presence of SbCl_3_: (a) with arylaldehydes, (b) with heteroaryl aldehydes.

Our investigations showed that *N,N*-dimethylaniline reacts smoothly with arylaldehydes and heterocyclic aldehydes in the presence of SbCl_3_ to produce the corresponding DTMs in good to excellent yields. At first we focused on the reaction of benzaldehyde and *N,N*-dimethylaniline as a model reaction under various reaction conditions (with solvent, solvent-free, and microwave-assisted). Various protic and aprotic solvents such as dimethyl sulfoxide, *N,N*-dimethylformamide, dichloromethane, ethanol, diethyl ether, and *n*-hexane were examined: The best results were obtained under solvent-free conditions without microwave irradiation. The general route for the synthesis of these compounds is shown in Scheme 1.

In a typical procedure, the reaction of benzaldehyde (0.50 mmol, 1 equiv) and *N*,*N*-dimethylaniline (1.25 mmol, 2.5 equiv) in the presence of SbCl_3_ (30 mol %) under solvent-free conditions at 120 °C for 4 h afforded compound **1a** in 80% yield (Table 1, entry 1). To determine the influence of different substituents on the generality of this reaction, a variety of aromatic aldehydes was examined (Table 1, entries 1–16). Furthermore, the number, nature, and position of these substituents affect the hue or color of the resulting dyes and the type of their applications.

**Table 1:** Synthesized diaminotriphenylmethanes (DTMs) **1a**–**1s** [39–45].

Entry	Product	Time (h)	Yield (%)^a^	Entry	Product	Time (h)	Yield (%)^a^

1	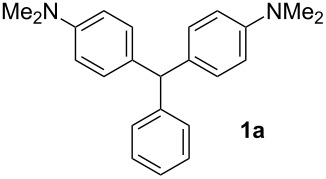	4	80	11	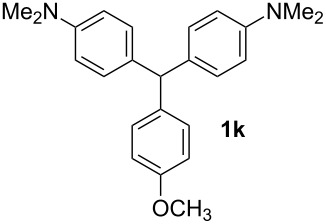	2	61
2	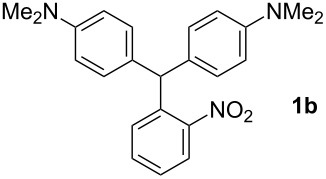	2	84	12	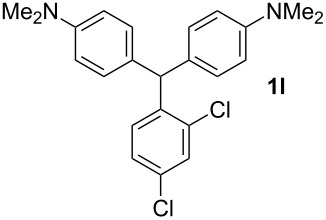	2	76
3	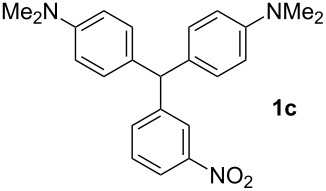	3	81	13	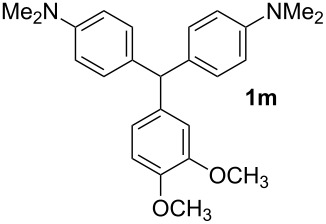	3	58
4	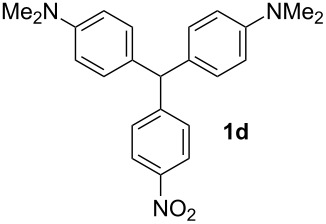	2	88	14	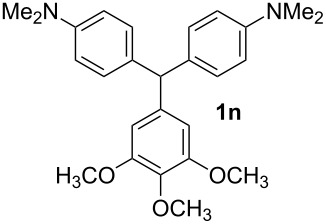	3	54
5	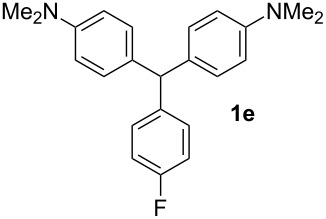	3	86	15	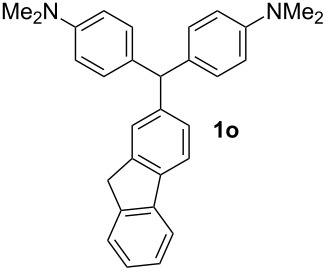	4	67
6	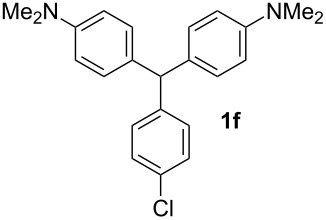	3	83	16	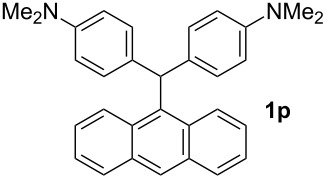	4	36
7	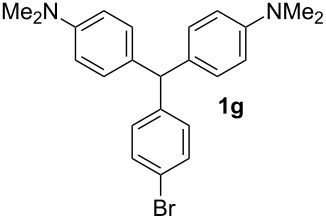	3	82	17	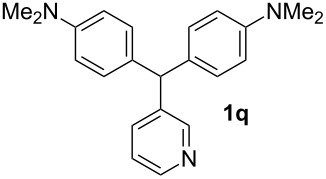	4	74
8	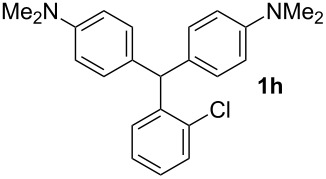	2	78	18	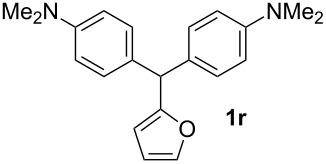	3	65
9	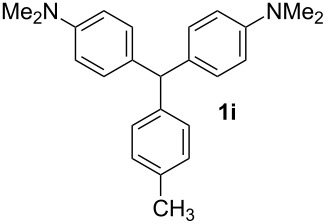	4	65	19	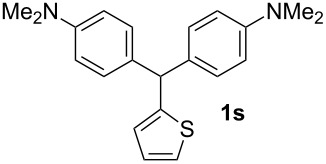	3	62
10	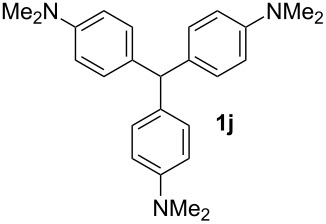	4	57				

^a^Isolated yield.

The dominant mechanism for the reaction can be summarized as a tandem regioselective electrophilic aromatic substitution reaction of *N,N*-dimethylaniline and aldehydes, in which SbCl_3_ (as a Lewis acid catalyst) activates the carbonyl group of the aldehydes. If we accept this mechanism, one can expect a general influence of electron-donating and electron-withdrawing groups on the feasibility of the reaction. Electron-withdrawing groups such as halo and nitro substituents at the *para*-position of the arylaldehydes increase the electrophilic strength of the carbonyl group and subsequently increase the yield of the reaction (Table 1, entries 4–7). An electron-withdrawing nitro-substituent at the *ortho*- or *meta*- position of benzaldehyde also gave good product yields (Table 1, entries 2–3). The presence of a nitro group in the *ortho*-position of benzaldehyde increases the inductive effect of NO_2_ but at the same time decreases its resonance effect due to its steric effect. The resonance effect of a *meta*-nitro group is relatively low and leads to a slightly lower yield of product in comparison to *ortho*-nitrobenzaldehyde. Thus, in these two cases, the yields are lower than with the *para*-nitro substituted benzaldehyde. On the other hand, electron-rich groups such as *para*-methoxy decrease the product yield (Table 1, entries 10–11). Surprisingly, benzaldehydes with two or even three deactivating methoxy groups react with *N,N*-dimethylaniline to produce the corresponding products in moderately good yields (Table 1, entries 13–14) which highlights the application of SbCl_3_ as useful catalyst in this methodology. Furthermore, the application range of the reaction was expanded to fused aromatic rings such as 9*H*-fluorene and anthracene: 9*H*-Fluorene-2-carbaldehyde and anthracene-9-carbaldehyde react with *N*,*N*-dimethylaniline under the above reaction conditions to afford the desired products **1o** and **1p** in yields of 67% and 36% yields, respectively (Table 1, entries 15–16). In the next step, the application of this procedure was further expanded to heterocyclic aldehydes for the synthesis of diaryl heteroaryl methane compounds (Scheme 1). These heterocyclic derivatives have received increasing attention due to their biological activities such as antibacterial and antitumor properties [44–45].

Both, electron-rich and electron-poor heterocyclic aldehydes including furfural, thiophene-2- and pyridine-3-carbaldehydes react with *N,N*-dimethylaniline under the same optimized reaction conditions to afford the corresponding 4,4’-diaminodiaryl heteroaryl methane compounds in relatively good yields (Table 1, entries 17–19).

### Computational details

**Figure 1:**
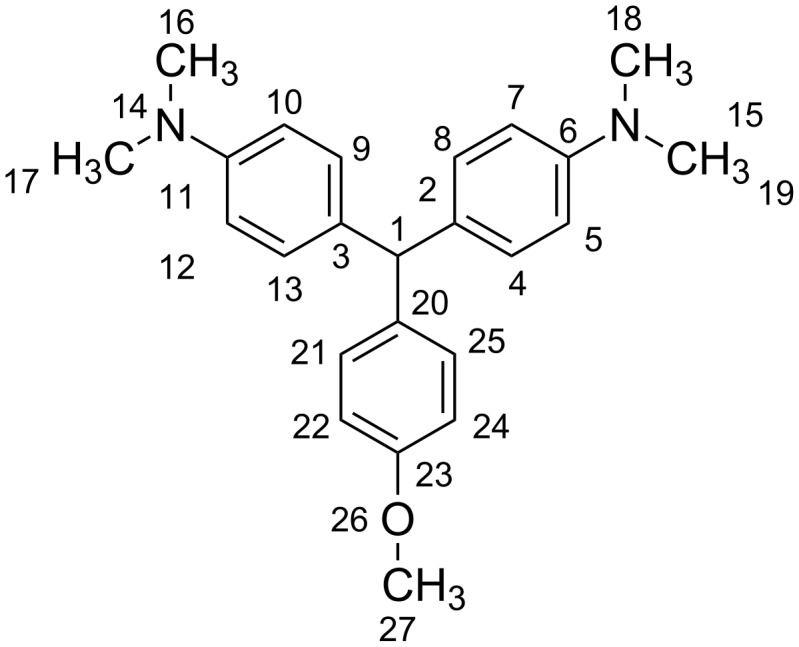
Molecular structure of compound **11**.

To predict the molecular structure of the title compounds and to assign their vibrational spectra, we performed theoretical calculations with compound **11** as the model compound (Figure 1). All theoretical calculations were carried out with the GAMESS program [46].

The first stage for geometry optimization of this molecule was completed by the standard HF/6-31G* method. The resulted HF geometry was then employed in further DFT calculations and re-optimization was carried out by 6-31G*/B3LYP and B3LYP/cc-pVDZ methods. The optimized structures were then used in the vibrational frequency calculations. The vibrational frequencies for these species were calculated and scaled by 0.899, 0.960, and 0.970 for HF/6-31G*, B3LYP/6-31G*, and B3LYP/cc-pVDZ methods, respectively. No imaginary frequency modes were obtained for the optimized structure of compound **11**, proving that a true minimum on the potential energy surface was found.

### The molecular structure of leuco compounds

**Figure 2:**
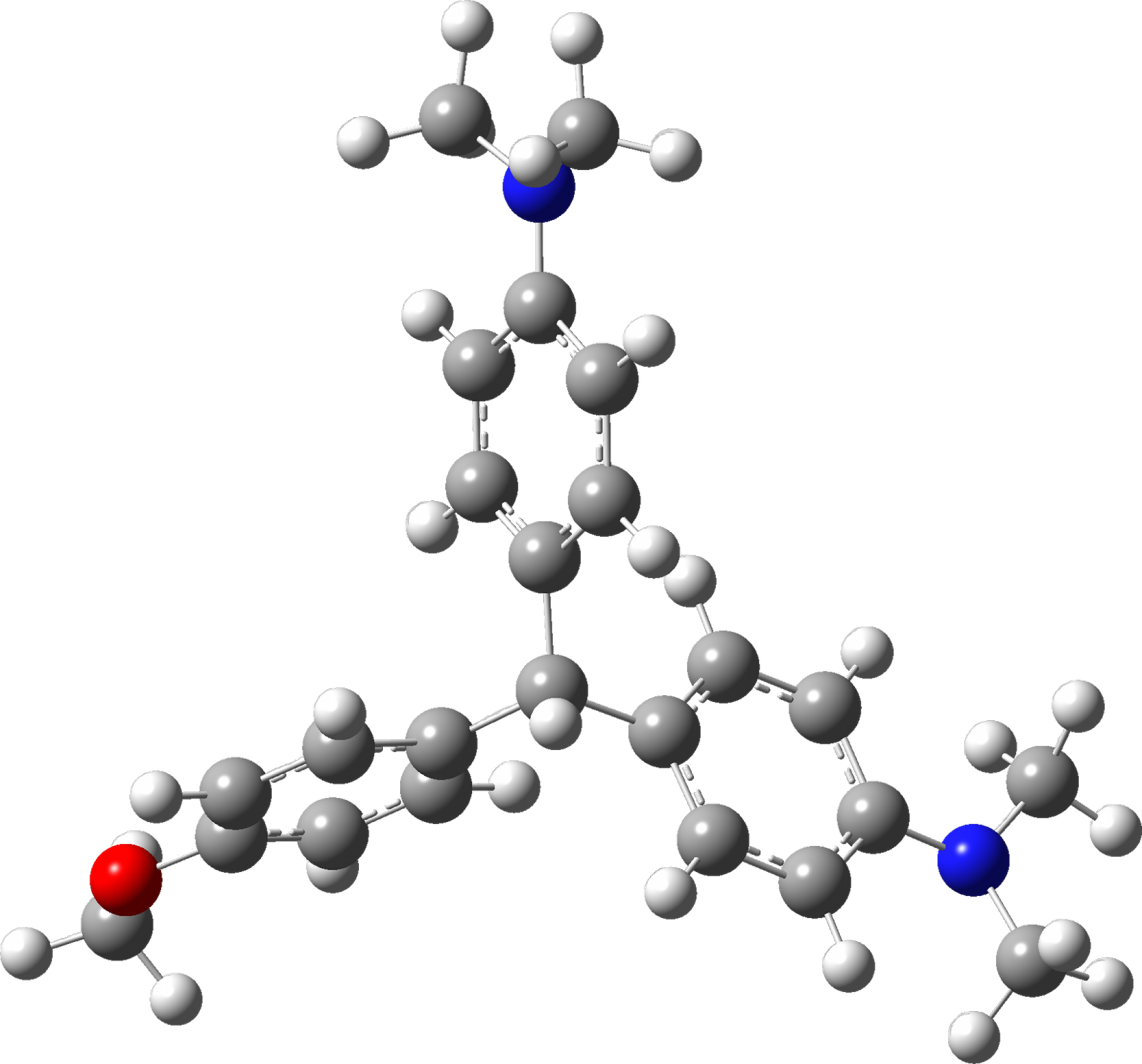
The molecular structure of compound **11** optimized by the B3LYP/cc-pVDZ method [47].

The optimized structural parameters of compound **11,** calculated by HF and DFT levels with the HF/6-31G*, 6-31G*/B3LYP, and B3LYP/cc-pVDZ methods, are listed in Table 2. The optimized configuration is shown in Figure 2. Because of unavailability of X-ray crystal structures for these compounds, the optimized structure is compared with X-ray structures of similar compounds. This comparison shows good agreement between optimized and actual molecular structures. Here, we compare the bond lengths of the C–C, C–O, and C–N bonds of similar structures with the results of HF/6-31G*, 6-31G*/B3LYP, and B3LYP/cc-pVDZ calculations. The optimized C–C bond lengths in the *N,N*-dimethylbenzenamine rings of compound **11** are in the range of 138.0–140.0 pm for the 6-31G*, 139.1–141.3 pm for the 6-31G*/B3LYP, and 139.3–141.5 pm for the B3LYP/cc-pVDZ calculations, which are in good agreement with a similar molecular structure in which these bond lengths are in the range of 136.5–140.5 pm [47]. The optimized C–C bond lengths in the anisole ring of compound **11** fall in the range of 137.3–139.8 pm for the 6-31G*, 138.7–140.5 pm for the B3LYP/6-31G*, and 138.8–140.5 pm for the B3LYP/cc-pVDZ calculations, which shows a good agreement with a similar molecular structure where the bond lengths are between 137.0 and 139.8 pm [48].

**Table 2:** The optimized geometrical parameters for compound **11**.

Parameters	HF/ 6-31G*	6-31G*/ B3LYP	B3LYP/ cc-pVDZ

Bond length (pm)			
C1–H1	110.201	110.336	110.319
C1–C2	153.050	153.188	153.164
C1–C3	152.841	153.016	153.005
C1–C20	153.084	153.398	153.361
C6–N15	141.685	141.353	139.428
C11–N14	139.574	139.584	139.411
C23–O26	135.024	136.774	136.779
O26–C27	139.755	141.779	141.676
Bond angles (°)			
C2–C1–C20	112.699	112.568	112.54
C3–C1–C20	113.353	113.395	113.444
C6–N15–C18	116.859	117.694	118.941
C6–N15–C19	115.066	117.357	118.907
C11–N14–C16	118.162	118.850	119.011
C11–N14–C17	118.057	118.796	119.017
C23–O26–C27	119.551	118.066	118.089
Dihedral angles (°)			
C1–C2–C8–C7	179.221	179.4578	178.960
C1–C2–C4–C5	178.147	178.777	178.759
C1–C3–C9–C10	179.235	179.229	179.182
C1–C3–C13–C12	179.053	179.387	179.424
C1–C20–C21–C22	179.273	178.632	178.864
C1–C20–C25–C24	179.475	178.831	179.053
C22–C23-O26–C27	0.06182	0.02378	0.41239
C24–C23–O26–C27	179.766	179.688	179.849

For the C23–O26 bond, the optimized bond lengths are 135.0 pm for the HF/6-31G* method, 136.7 pm for the B3LYP/6-31G* method, and 136.7 pm for the B3LYP/cc-pVDZ calculations which is in good agreement with a bond length of 137.0 pm found in similar compounds [48].

The bond lengths of C11–N14 and C6–N15 are 139.5 pm and 141.6 pm for the HF\6-31G*, 139.5 pm and 141.3 pm for the B3LYP/6-31G*, and 139.4 pm and 139.4 pm for the B3LYP/cc-pVDZ calculations. As one can see, these bond lengths are in good agreement with the X-ray results obtained for similar compounds where the bond lengths are 137.8 and 140.0 pm [49]. Although there are some differences between our results and those from X-ray structure analysis of similar compounds, the optimized structural parameters are very close to the X-ray values and are good enough for further vibrational calculations.

### Assignments of vibrational frequencies

**Figure 3:**
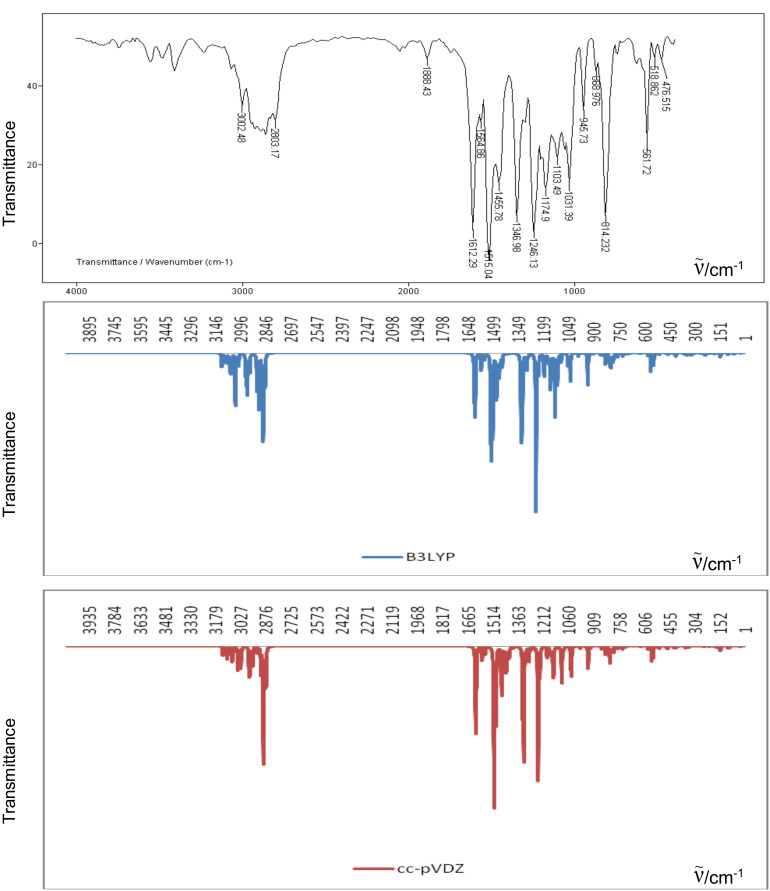
Experimental infrared spectrum of compound **11** and infrared spectrum calculated with B3LYP and cc-pVDZ methods.

To our best knowledge, no vibrational data and assignments of the modes have been reported for leuco compounds. The experimental infrared spectrum and the calculated infrared spectra of **11** are shown in Figure 3.

**Figure 4:**
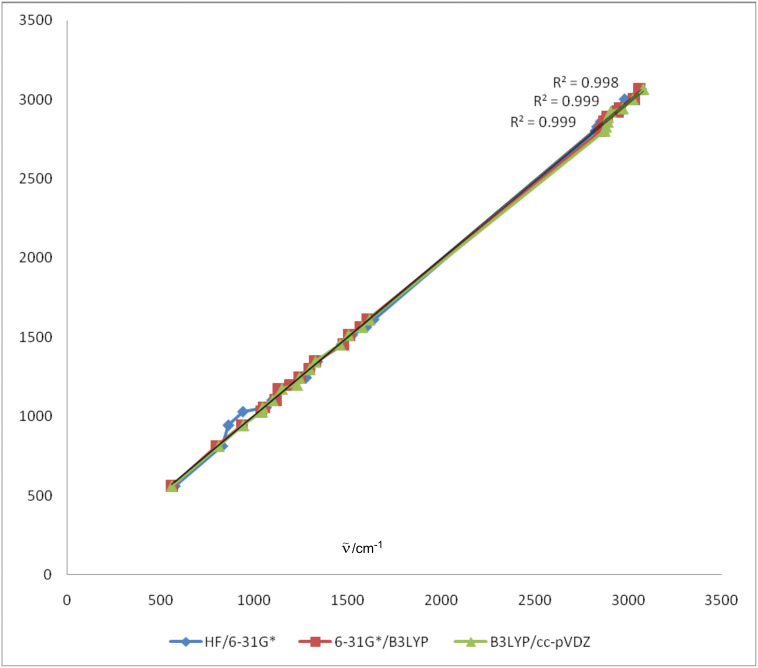
Linear correlation between the experimental and the theoretical frequencies (cm^−1^) obtained by HF/6-31G*, 6-31G*/B3LYP/cc-PVDZ methods.

A comparison between the infrared spectra of this compound can be made by establishing a correlation between the observed and the theoretically calculated wavenumbers. Linear correlations between the experimental and calculated wave numbers have been found (Figure 4). The correlation coefficients indicate a good linearity between the calculated and experimental wavenumbers. These correlation coefficients are 0.998, 0.999, and 0.999 for HF/6-31G*, 6-31G*/B3LYP, and B3LYP/cc-pVDZ methods, respectively.

Based on this comparison between the calculated and experimental IR spectrum, assignments of the fundamental modes were carried out on the basis of the B3LYP/cc-pVDZ calculations.

The resulting vibrational wavenumbers for the optimized geometry and the proposed assignments are given in Table 3. The observed bands at 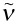 = 3067, 3003, 2945, 2924, 2890, 2861, 2829, and 2803 cm^−1^ are assigned to the aromatic CH and aliphatic CH_3_ stretching modes, whereas the C–H stretching vibrations in aromatic rings occur above 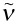 = 3000 cm^−1^. The principal bands in the 1600–1000 cm^−1^ region are the C–O and C–N stretching vibrations modes as well as the bending vibrations of C–H bonds (Table 3).

**Table 3:** Comparison of the observed and the calculated vibrational wavenumbers (cm^−1^) of compound **11** (ν, stretching; δ, in-plane bending; π, out-of-plane bending; ω, wagging. Subscript: asym: asymmetric; sym: symmetric).

	EXP	HF	B3LYP	cc-pVDZ	Assignment

1	561.576	574.84	559.79	559.73	π (rings)
2	814.226	829.10	798.49	810.33	π (ring)
3	945.731	861.27	934.31	941.38	*ν*_sym_ (C–N)
4	1031.39	938.77	1037.28	1041.51	*ν* (C–O)
5	1058.06	1062.23	1051.95	1045.08	π (C–H of N(CH_3_)_2_
6	1103.49	1094.70	1114.83	1099.25	π (C–H of N(CH_3_)_2_
7	1174.91	1147.57	1128.25	1148.25	δ (C–H of anisole ring)
8	1200.32	1212.88	1192.91	1229.80	δ (C–H)
9	1246.14	1276.49	1241.33	1241.87	*ν* (C–O)
10	1299.39	1296.32	1293.91	1296.63	δ (C–H of Ph rings)
11	1346.98	1336.18	1325.74	1326.77	*ν* (C–N)
12	1455.78	1466.38	1476.46	1458.62	π (C–H of N(CH_3_)_2_)
13	1515.04	1523.14	1506.76	1504.60	δ (C–H of Ph rings)
14	1564.87	1598.21	1566.91	1577.69	δ (C–H of anisole ring)
15	1612.29	1637.60	1604.45	1616.06	δ (C–H of anisole ring)
16	2803.27	2832.43	2848.99	2872.77	*ν*_sym_ (C–H of N(CH_3_)_2_)
17	2829.10	2832.43	2860.60	2880.32	*ν*_sym_ (C–H of N(CH_3_)_2_)
18	2860.91	2851.48	2860.60	2889.46	*ν*_sym_ (C–H of N(CH_3_)_2_)
19	2889.90	2881.90	2884.14	2889.82	*ν*_sym_ (C–H of N(CH_3_)_2_)
20	2923.99	2926.87	2897.76	2904.77	*ν*_sym_ (C–H of OCH_3_)
21	2944.57	2935.79	2953.94	2969.29	*ν*_asym_ (C–H of N(CH_3_)_2_)
22	3002.6	2979.15	3026.16	3024.22	*ν*_asym_ (C–H of N(CH_3_)_2_)
23	3066.86	3067.33	3056.959	3079.631	*ν*_asym_ (C–H of anisole ring)

Some vibrational frequencies over 2900 cm^−1^ are not observed clearly in the experimental spectra whereas they are obtained from DFT calculations. For instance, the peaks at 2951, 2955 and 3042 cm^−1^ are for 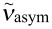 [C–H of N(CH_3_)_2_] vibrations in the theoretical spectra (there is also a peak in 2976 cm^−1^ for 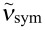 [C–H of N(CH_3_)_2_] in the calculated infrared spectra). Other peaks at 2971, 3039 cm^−1^ (for 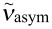 C–H of OCH_3_), 3130 and 3134 cm^−1^ (C–H symmetric stretching vibrations) are also not discernable in the experimental IR spectrum.

## Conclusion

In summary, it has been demonstrated that SbCl_3_ is a mild and efficient catalyst for the one-pot reaction of *N,N*-dimethylaniline with a variety of aryl and heteroaryl aldehydes under solvent-free conditions to give substituted triarylmethanes. Using SbCl_3_ as catalyst, even electron-rich benzaldehydes such as 3,4,5-trimethoxybenzaldehyde gave the corresponding products in good yields. Operational simplicity, high yields, and the ability to prepare a wide range of products are the advantages of this protocol. Molecular geometry parameters and vibrational wavenumbers of the triarylmethanes have been obtained from theoretical calculations for the first time. The theoretical results show good agreement between theoretical and experimental data.

## Experimental

### General

Commercial grade aldehydes and *N,N*-dimethylaniline were purchased from Merck or Aldrich. The solvents were of analytical grade and were used as received. Silica gel (Merck, grade 9385, 230–400 mesh, 60 Å) for column chromatography was used as received. The course of the synthesis and the purity of the products were monitored by TLC on silica gel plates (Merck F_60_ 254, 20110, 0.2 mm, ready-to-use), with ethyl acetate/*n*-hexane (1:4) as eluent. The eluent for PTLC was the same as the TLC eluent. Melting points were determined with a Gallenkamp melting point apparatus and a Kofler hotplate, and are uncorrected. ^1^H and ^13^C NMR spectra were recorded with either a Bruker AC300 or a 500 MHz spectrometer at ambient temperature. ^1^H NMR spectra are referenced to tetramethylsilane (0.00 ppm) and ^13^C NMR spectra are referenced to residual solvent peaks (for example, 77.23 ppm for CDCl_3_). Chemical shifts are given in ppm. Infrared absorption spectra were obtained using a Shimadzu 4300 FTIR spectrometer as thin films between potassium bromide plates. IR is reported as characteristic bands (cm^−1^) at their maximum intensity. Broad signals are denoted as br. Elemental analyses were carried out with a Heareus CHN-RAPID instrument.

#### Typical procedure for the synthesis of of 4,4'-diaminotriarylmethane derivatives

A vial equipped with a stirring bar was charged with the arylaldehyde (0.5 mmol, 1.0 equiv), *N,N*-dimethylaniline (1.25 mmol, 2.5 equiv), and SbCl_3_ (30 mol %) and the vial was capped. The resulting mixture was heated in an oil bath at 120 °C for an appropriate time (Table 1). The progress of the reaction was monitored by TLC. Then the reaction mixture was cooled to room temperature, diluted with dichloromethane and filtered. The filtrate was concentrated in vacuo and the residue purified by crystallization or column chromatography on silica gel (ethyl acetate/*n*-hexane). The spectral data and elemental analysis for selected products are listed below.

#### 3,4-Dimethoxyphenyl-bis[4-(dimethylamino)phenyl]methane (1m)

Yield: 58%; Colorless oil. IR (KBr): 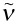 = 3007, 2896, 1618, 1515, 1455, 1340, 1246, 1175, 814 cm^−1^. ^1^H NMR (300 MHz, CDCl_3_, ppm): *δ* = 2.94 (s, 12H), 3,79 (s, 3H), 3.87 (s, 3H), 5.35 (s, 1H), 6.64 (dd, *J =* 1.5 Hz, 8.2 Hz, 1H), 6.71 (m, 5H), 6.79 (d, *J* = 8.2, 1H), 7.01 ppm (d, *J* = 8.8, 4H). ^13^C NMR (75 MHz, CDCl_3_, ppm): *δ* = 41.3, 55.0, 56.2, 56.3, 111.2, 113.1, 113.2, 121.7, 130.3, 133.8, 138.5, 147.6, 149.1, 149.3 ppm. Anal. Calcd. for C_25_H_30_N_2_O_2_ (390.5): C 76.89; H 7.74; N 7.17. Found: C 76.98; H 7.82; N 7.24.

#### 3,4,5-Trimethoxyphenyl-bis[4-(dimethylamino)phenyl]methane (1n)

Yield: 54%; Colorless oil. IR (KBr): 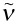 = 3004, 2891, 1612, 1541, 1348, 1290, 1183, 1031, 946, 817 cm^−1^. ^1^H NMR (300 MHz, CDCl_3_, ppm): *δ* = 2.97 (s, 12H), 3,75 (s, 6H), 3.84 (s, 3H), 5.33 (s, 1H), 6.35 (s, 2H), 6.78 (d, *J =* 8.6, 4H), 7.02 ppm (d, *J =* 8.6, 4H). ^13^C NMR (75 MHz, CDCl_3_, ppm): *δ* = 30.9, 55.3, 56.1, 60.8, 106.5, 130.0, 135.3, 137.5, 139.2, 148.4, 152.9, 153.6 ppm. Anal. Calcd. for C_26_H_32_N_2_O_3_ (420.5): C 74.26; H 7.67; N 6.66. Found: C 74.39; H 7.66; N 6.78.

#### (*9H*)-Fluoren-2-yl-bis[4-(dimethylamino)phenyl]methane (1o)

Yield: 67%; Colorless crystals; mp 184–185 °C. IR (KBr): 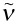 = 3006, 2875, 1612, 1516, 1479, 1447, 1346 cm^−1^; ^1^H NMR (500 MHz, CDCl_3_, ppm): *δ* = 2.94 (s, 12H), 3.84 (s, 2H), 5.49 (s, 1H), 6.72 (d, *J* = 8.4 Hz, 4H), 7.06 (d, *J* = 8.4 Hz, 4H), 7.19 (d, *J* = 7.8 Hz, 1H), 7.27 (m, 1H), 7.36 (m, 2H), 7.52 (d, *J* = 7.5 Hz, 1H), 7.69 (d, *J* = 7.8 Hz, 1H), 7.76 ppm (d, *J* = 7.5 Hz, 1H). ^13^C NMR (125 MHz, CDCl_3_, ppm): *δ* = 37.4, 41.3, 55.6, 113.1, 119.9, 120.1, 125.4, 126.4, 126.7, 127.1, 128.6, 130.5, 133.6, 140, 142.2, 143.7, 143.8, 144.8, 149.3 ppm. Anal. Calcd. for C_30_H_30_N_2_ (418.5): C 86.08; H 7.22; N 6.69. Found: C 86.19; H 7.15; N 6.75.

#### Anthracen-9-yl-bis[4-(dimethylamino)phenyl]methane (1p)

Yield: 36%; Colorless crystals; mp 68–69 °C. IR (KBr): 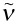 = 3011, 2835, 1612, 1521, 1449, 1346 cm^−1^; ^1^H NMR (500 MHz, CDCl_3_, ppm): *δ* = 2.85 (s, 6H), 2.96 (s, 6H), 5.13 (s, 1H), 6.61 (m, 4H), 6.98 (m, 2H), 7.18 (m, 3H), 7.28 (m, 4H), 7.35 (m, 2H), 7.55 (d, *J* = 7.6 Hz, 1H), 7.75 ppm (d, *J* = 7.6 Hz, 1H). ^13^C NMR (125 MHz, CDCl_3_, ppm): *δ* = 40.9, 52.2, 112.4, 123.9, 125.7, 126.9, 127.2, 127.5, 128.3, 128.5, 128.8, 129.1, 129.5, 130.2, 130.7, 135.3, 139.6, 141.8 ppm. Anal. Calcd. for C_31_H_30_N_2_ (430.5): C 86.47; H 7.02; N 6.51. Found: C 86.59; H 7.09; N 6.58.
